# Understanding the patterns of antibiotic susceptibility of bacteria causing urinary tract infection in West Bengal, India

**DOI:** 10.3389/fmicb.2014.00463

**Published:** 2014-09-18

**Authors:** Sunayana Saha, Sridhara Nayak, Indrani Bhattacharyya, Suman Saha, Amit K. Mandal, Subhanil Chakraborty, Rabindranath Bhattacharyya, Ranadhir Chakraborty, Octavio L. Franco, Santi M. Mandal, Amit Basak

**Affiliations:** ^1^Microbiology Laboratory, Department of Biological Sciences, Presidency UniversityKolkata, India; ^2^Department of Chemistry, Central Research Facility, Indian Institute of Technology KharagpurKharagpur, India; ^3^Department of Microbiology, Calcutta School of Tropical MedicineKolkata, India; ^4^Ocular Microbiology & Molecular Biology Laboratory, Priyamvada Birla Aravind Eye HospitalKolkata, India; ^5^Department of Biotechnology, North Bengal UniversitySiliguri, India; ^6^Department of Microbiology, Vidyasagar UniversityMidnapore, India; ^7^Centro de Análises Proteômicas e Bioquímicas, Pós-Graduação em Ciências Genômicas e Biotecnologia, Universidade Católica de BrasíliaBrasilia, Brazil; ^8^Pós-Graduação em Biotecnologia, Universidade Católica Dom BoscoCampo-Grande, Brazil

**Keywords:** antibiotic susceptibility, uropathogens, West Bengal, India, pathogenic bacteria

## Abstract

Urinary tract infection (UTI) is one of the most common infectious diseases at the community level. In order to assess the adequacy of empirical therapy, the susceptibility of antibiotics and resistance pattern of bacteria responsible for UTI in West Bengal, India, were evaluated throughout the period of 2008–2013. The infection reports belonging to all age groups and both sexes were considered. *Escherichia coli* was the most abundant uropathogen with a prevalence rate of 67.1%, followed by *Klebsiella* spp. (22%) and *Pseudomonas* spp. (6%). Penicillin was least effective against UTI-causing *E. coli* and maximum susceptibility was recorded for the drugs belonging to fourth generation cephalosporins. Other abundant uropathogens, *Klebsiella* spp., were maximally resistant to broad-spectrum penicillin, followed by aminoglycosides and third generation cephalosporin. The antibiotic resistance pattern of two principal UTI pathogens, *E. coli* and *Klebsiella* spp. in West Bengal, appears in general to be similar to that found in other parts of the Globe. Higher than 50% resistance were observed for broad-spectrum penicillin. Fourth generation cephalosporin and macrolides seems to be the choice of drug in treating UTIs in Eastern India. Furthermore, improved maintenance of infection incident logs is needed in Eastern Indian hospitals in order to facilitate regular surveillance of the occurrence of antibiotic resistance patterns, since such levels continue to change.

## INTRODUCTION

The increasing emergence of bacterial resistance to a large number of antibiotics is causing major health concerns worldwide. Nowadays, infectious pathogens are mostly resistant to several antibiotics, and this undermines the ability of antibiotics to control infections (Tseng et al., 2008; Haider et al., 2010; Bahadin et al., 2011; Nerurkar et al., 2012; Sood and Gupta, 2012). Urinary tract infection (UTI), either hospital acquired or community acquired, is one of the most common infections. An estimate of patients suffering from UTI is around 150 million per annum across the Globe, which may rise to 75% in the female population by the age of 24, and 15–25% of this group will suffer from a relapse of this disease (Hooton, 2001; Foxman, 2002; Hunstad, 2010; Mukherjee et al., 2013; Pouwels et al., 2013). The antimicrobial resistance epidemiological survey on cystitis (ARESC), an international survey on antimicrobial resistance of pathogens, demonstrated that *E. coli* showed high resistance to sulfonamide SXT (double-strength trimethoprim-sulfamethoxazole) and to fluoroquinolone ciprofloxacin in nine European countries and in Brazil (Schito et al., 2009). The occurrence of SXT resistance among urinary pathogens appears extensive in the United States, and it seems to be expected that SXT, sooner or later, will need to be replaced by alternative therapies (Gupta et al., 2001). Despite the fact that UTI is the third most common infection found in India, only a few studies on UTI in this country have been reported (Akram et al., 2007; Kothari and Sagar, 2008; Hussain et al., 2012; Prakash and Saxena, 2013). In eastern India, UTI is a common infection found among all ages from infants to elderly persons. However, studies on UTI and the susceptibility pattern of antibiotics in Eastern India are still underway, and there is extensive debate on the choice of antibiotics due to the lack of clear guidelines. Knowledge of the etiology and antibiotic susceptibility pattern of the pathogen causing UTI is absolutely essential. In the global as well as the national context, uropathogens treated empirically with antibiotics have been regarded as a potential cause for the emergence of antibiotic resistance among several classes of bacteria. Although empirical antimicrobial treatment for UTI is accepted clinically, bacteria are developing resistance to antibiotics faster than the development of new classes of antibiotics. Very often clinicians prescribe broad-spectrum antibiotics instead of a specific antibiotic during empirical treatment, in view of the resistance of the causative organism against the antibiotic. Antibiotic misuse and patients who are non-compliant or who do not complete the course of antibiotic therapy cause an increase in antibiotic-resistant bacteria.

In this present study, the trends of the antibiotic-resistant uropathogens and their susceptibility toward various antibiotics are considered. This study will further help in formulating guidelines for establishing a proper empirical therapy for UTIs while awaiting culture sensitivity reports. It also reflects changes in the susceptibility pattern of some of the most common uropathogens over the years, in Eastern India, implying the need for periodic monitoring in order to decrease the number of therapeutic failures and to boost an effort to arrest the growing occurrence of antibiotic resistance.

## MATERIALS AND METHODS

### STUDY DESIGN AND AREA

The data were taken from the laboratory register of the School of Tropical Medicine, Sanjiban Hospital, Uluberia; North Bengal University Health Centre, Darjeeling; and several private hospitals in Paschim Medinipur and Hooghly districts from January 2008 to July 2013. The anonymity of the patients was ensured. All data were retrospectively collected and de-identified when this was necessary to ensure patient confidentiality. The study included all the patients who were admitted or visited the out-patient department in the hospital or health centre with symptoms of UTI during the study period and then had UTI confirmed further by positive urine culture reports. Patients who underwent treatment with another antimicrobial within 48 h or within 24 h, receiving only a single dose and in the presence of an appropriate positive culture, were also excluded from the study.

### ISOLATION AND IDENTIFICATION OF UROPATHOGENS

A clean-catch midstream specimen was collected in a sterile wide-mouth leak-proof container to hold about 50 ml specimens. Using the calibrated loop method with a loop diameter of 4 mm, 10 μl of uncentrifuged specimen was transferred onto the agar plate and streaked without flaming the loop, for isolation, and incubated at 35–37°C for 24 h. A specimen was considered positive for UTI when the density of the bacterium was ≥10^5^ colony-forming units (CFU)/ml. The single-colony type cultures were identified using standard microbiological methods up to genus/species levels wherever applicable.

### ANTIBIOTIC SENSITIVITY TESTING

Antibiotic sensitivity testing was done following the Kirby-Bauer disc diffusion method according to the Clinical and Laboratory Standards Institute (CLSI) guidelines. The antibiotics tested were broad-spectrum penicillin, third generation Cephalosporin, fourth generation Cephalosporin, Quinolones, Tetracycline, Macrolides, Aminoglycosides, and Sulfonamides (Himedia, India).

### STATISTICAL ANALYSIS

The methodology used in this study was based on the controlled cluster technique. The technique was used to classify the percentage distributions of resistant capacity of the drugs into a number of categories. The lowest percentage, 4% in this case, was taken as susceptible and the highest (59%) was taken as highly resistant. Windows XP based ArcGIS version 9.3 was used for the categorization. This was applied to all the drugs used in this study for all the 6 years 2008–2013. The polynomial trendlines, except tetracycline, were also computed to study the nature of the resistant capacity of each drug. These polynomial trendlines were calculated depending on the nature of the curve drawn from the percentage distribution of each drug in 6 years. Linear trend was computed in the case of tetracycline. A number of polynomial trendlines with different orders starting from 2 to 5 were generated using Microsoft Excel 2003 until the best fitting trendline was found for the nature of each drug. The main purpose of doing this was to demonstrate the overall pattern of the data, whether it was going up or down, and to predict the data during these 6 years and what may happen in the future. Finally, radar plots were made to compare the efficiency of each drug as a whole during 2008–2013. These plots were also generated, through Microsoft Excel 2003, mainly to investigate the relative strength and relative weakness in different years, as well as to depict overall performance.

## RESULTS

**FIGURE 1:**
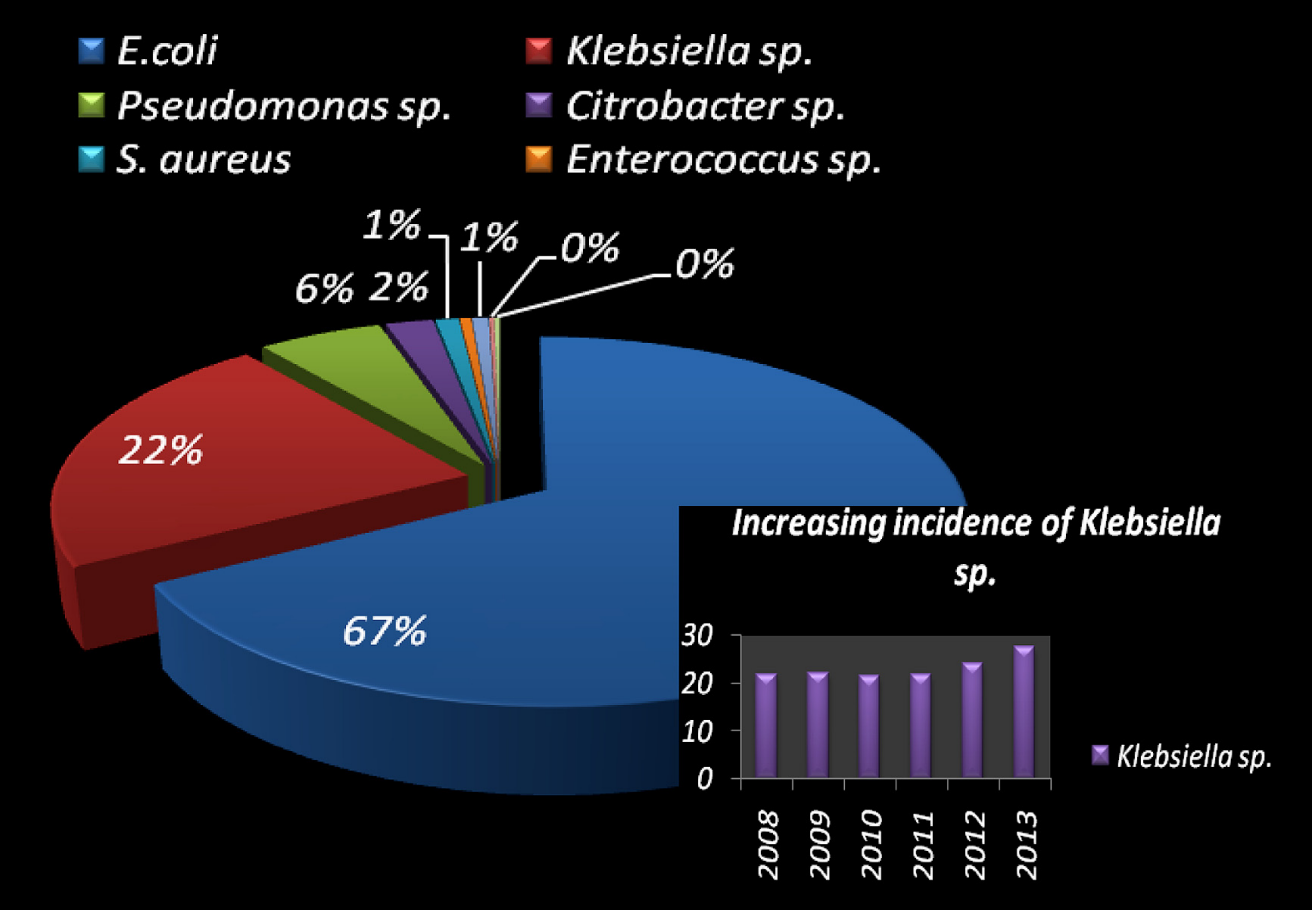
**Pie-chart visualization of total bacterial species, isolated in urinary tract infected patient in West Bengal, India.** Distribution (in %) of the most frequent uropathogens and increasing incidence of *Klebsiella* spp. causing UTI (in inset).

**FIGURE 2:**
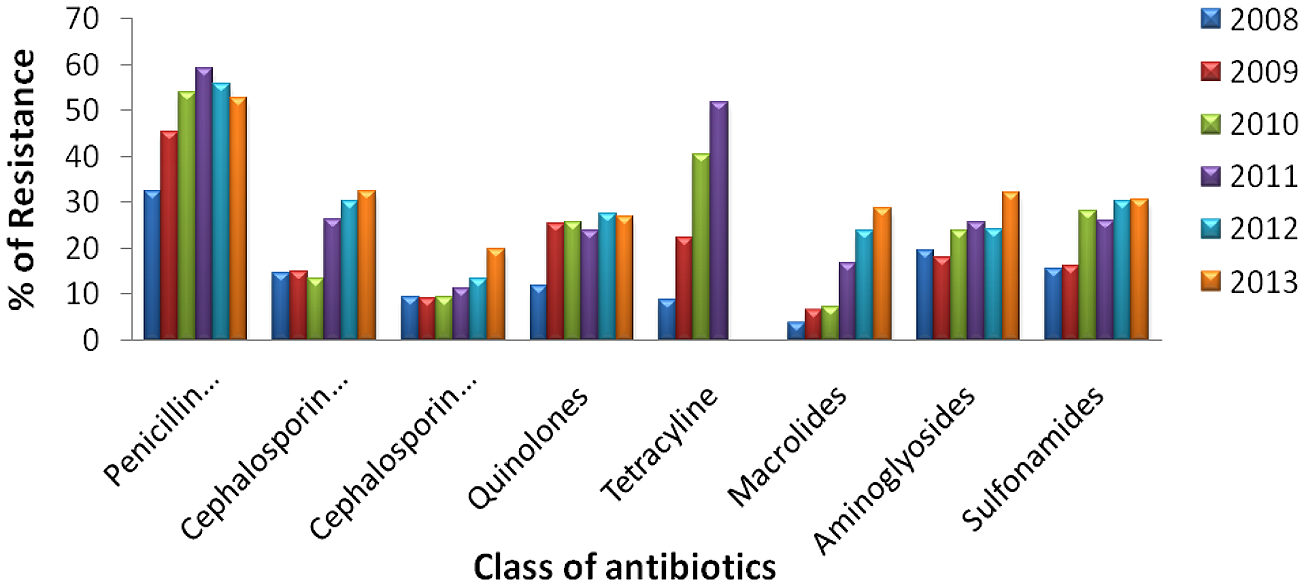
**Antibiotic resistance pattern of *E. coli.*** Change of resistance pattern of different antimicrobials in *E. coli* over the last 6 years (from 2008 to 2013).

**FIGURE 3:**
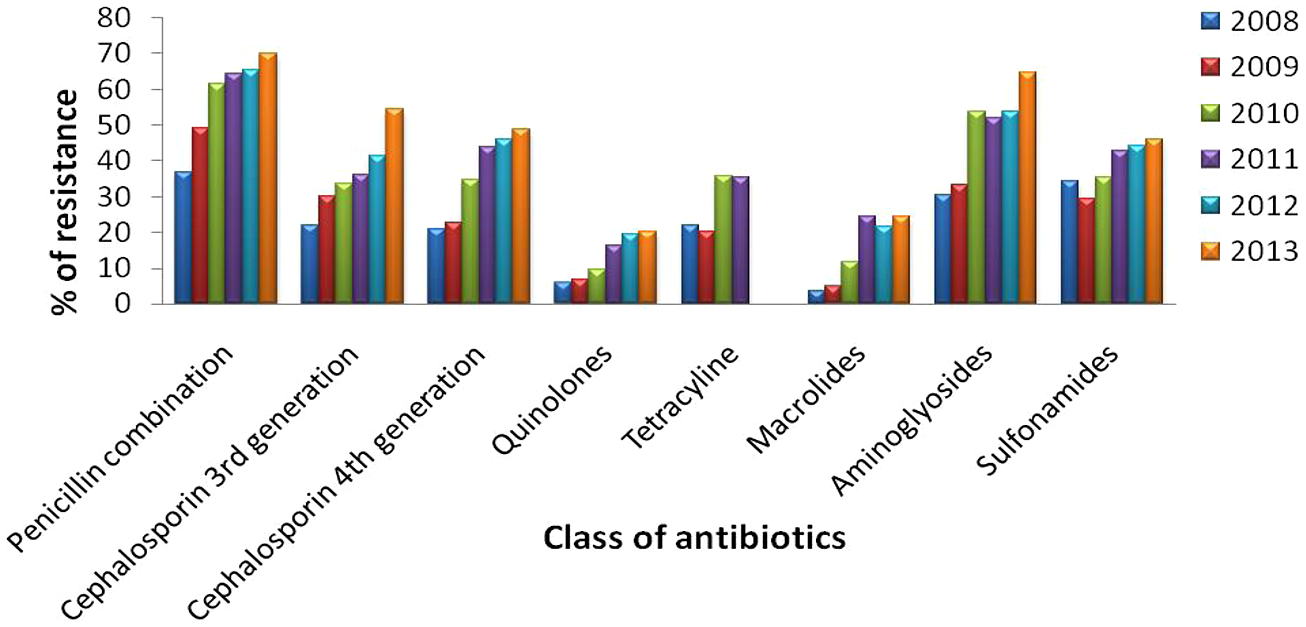
**Antibiotic resistance pattern of *Klebsiella* spp.** Change of resistance pattern of different antimicrobials in *Klebsiella* spp. over the last 6 years (from 2008 to 2013).

Urinary tract infections mostly occurred among 2- to 13- year-old girls and 7 million acute uncomplicated infections occurred annually among young women only in USA (Schappert, 1992). Catheter-associated UTIs account for 40% of all hospital-acquired infections and are the most common type of nosocomial infection. UTIs are an important reservoir for selection and transmission of multi-drug-resistant strains (Kunin, 1997; Stamm, 1999). In this study, the antibiotic susceptibility data of consecutive samples from positive UTIs in a period of 6 years (Jan 2008 to June 2013) from an eastern state of India were analyzed. All the acquired data of 19 antibiotics were classified, – segregated properly as broad-spectrum penicillin, third generation Cephalosporin, fourth generation Cephalosporin, Quinolones, Tetracycline, Macrolides, Aminoglycosides, and Sulfonamides. The infection report belonged to all age groups, and both sexes were reviewed in this study. Of the total culture samples (5476) analyzed, *E. coli* was the most dominant among all isolated uropathogens with a prevalence rate of 67.1%, followed by *Klebsiella* spp. (22%), *Pseudomonas* spp. (6%), *Citrobacter* spp. (2%), *Staphylococcus aureus* (1%), and *Enterococcus* spp. (1%) [**Figure 1**]. Interestingly, *Klebsiella* spp. infection in Eastern India is increasing rapidly with time (**Figure 1**, inset). *E. coli* causes 80–90% of acute uncomplicated bacterial lower tract infections (cystitis) in young women. It has been estimated that up to 15% of patients with UTI are bacteremic, and that *E. coli* was the predominant pathogen of bacteremic gram-negative UTI, followed by *Klebsiella pneumoniae.* The change in *E. coli* resistance patterns to different antibiotics over the last 6 years (from 2008 to 2013) is shown in **Figure 2**, and the second predominant causal organism, *Klebsiella* spp. for UTI in India, demonstrates the gradation of resistance to different groups of antibiotics, as reflected in **Figure 3**.

**FIGURE 4:**
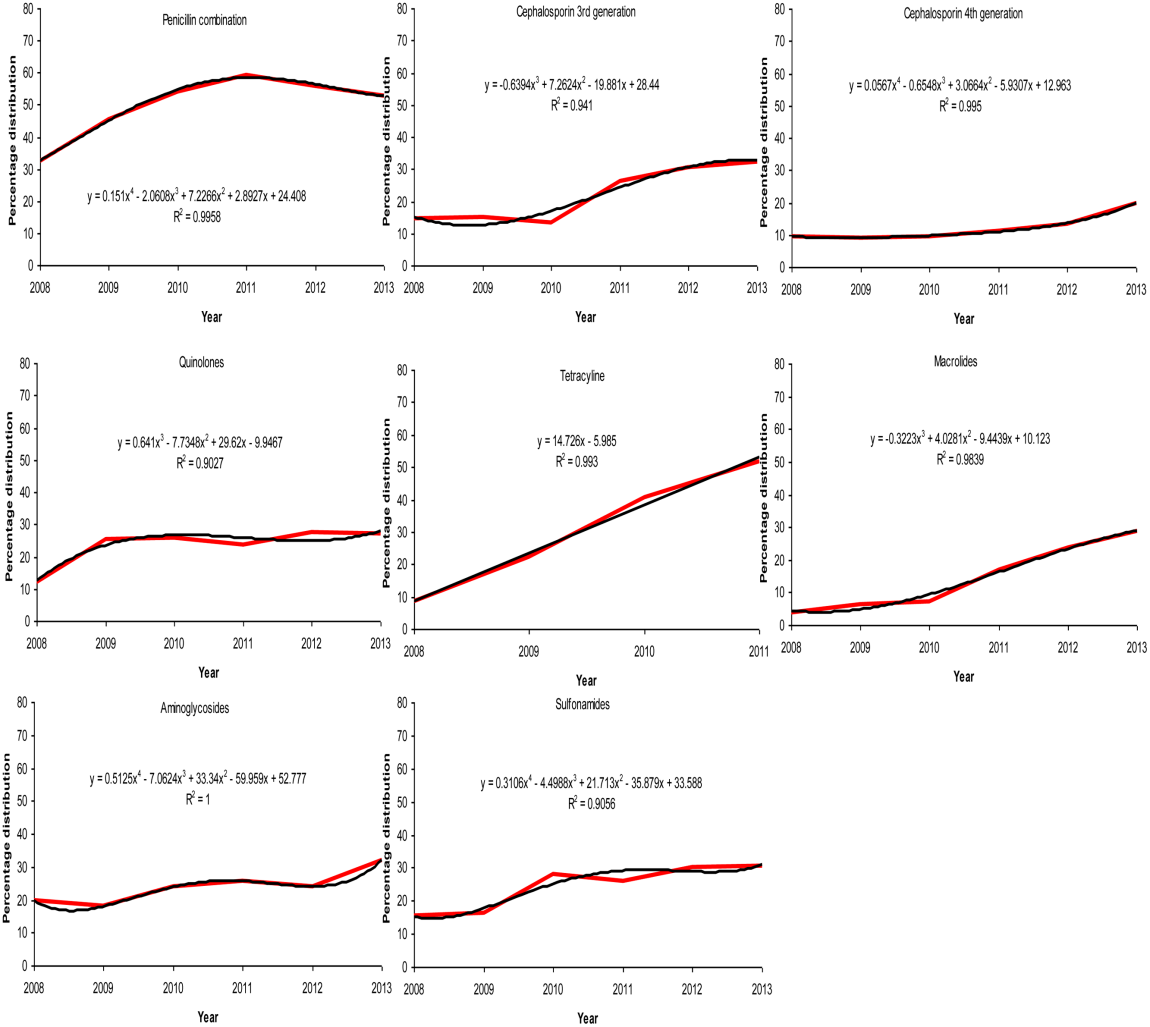
**Trend analyses of the development of resistance to different antibiotics**.

The behavior of each drug during the period 2008–2013 is presented in **Figure 4**. The equations in each sub-figure show the trendline and the coefficient of determination (*R*^2^) of the corresponding drug. Polynomial regression is generally used to describe non-linear phenomena; in our case we wanted to test the phenomenon of progression of resistance to certain groups of antibiotics over time. It is interpreted that a trendline is most reliable when the *R*^2^ value is at 1 or near 1. Since all the *R*^2^ values computed are >0.9, the values are not random and present a good fit to the proposed equation. This shows that a polynomial trendline of order 4 is found to be fitted for a penicillin combination, fourth generation cephalosporin, aminoglycosides and sulfonamides, while the same of order 3 is found to be fitted for third generation cephalosporin, quinolones, and macrolides.

**FIGURE 5:**
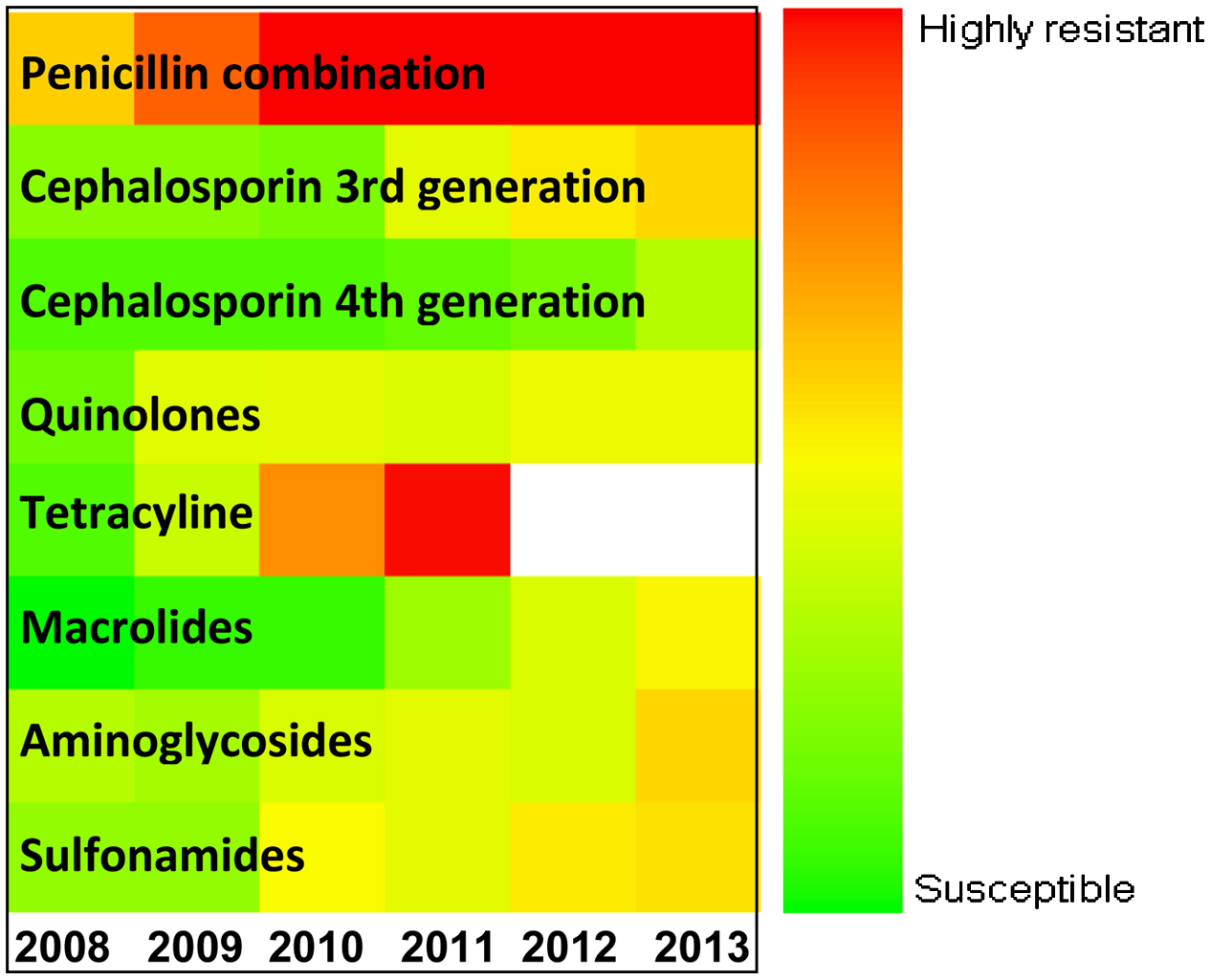
**Antibiotic sensitivity/resistance in major uropathogens of Eastern India (from 2008 to 2013).** Color spectrum from green to red indicates progressive increase in resistance. Green denotes susceptible; Red denotes alarmingly high degree of resistance.

**Figure 5** explains the drug panel with a varying degree of therapeutic potential in the face of emerging antibiotic resistance. The red color indicates the drug candidate being least effective because of high incidence of resistance shown by the uropathogens, while the green colored drugs are the ones to which the pathogens are most susceptible. It was found that the penicillin combination is least effectual, while the fourth generation cephalosporin antibiotics are most effective. Overall use of tetracycline showed a steady rise in the resistance pattern for both the uropathogens during the study period, and use of this drug has been falling since 2010. The resistance pattern toward quinolones was the same from 2008 to 2013 for both the *E. coli* and *Klebsiella* spp. isolates. Fourth generation cephalosporins have been found to be the most effective against both the pathogens. However, a slight increase in cephalosporin-resistant uropathogens was noted in the year 2013.

**FIGURE 6:**
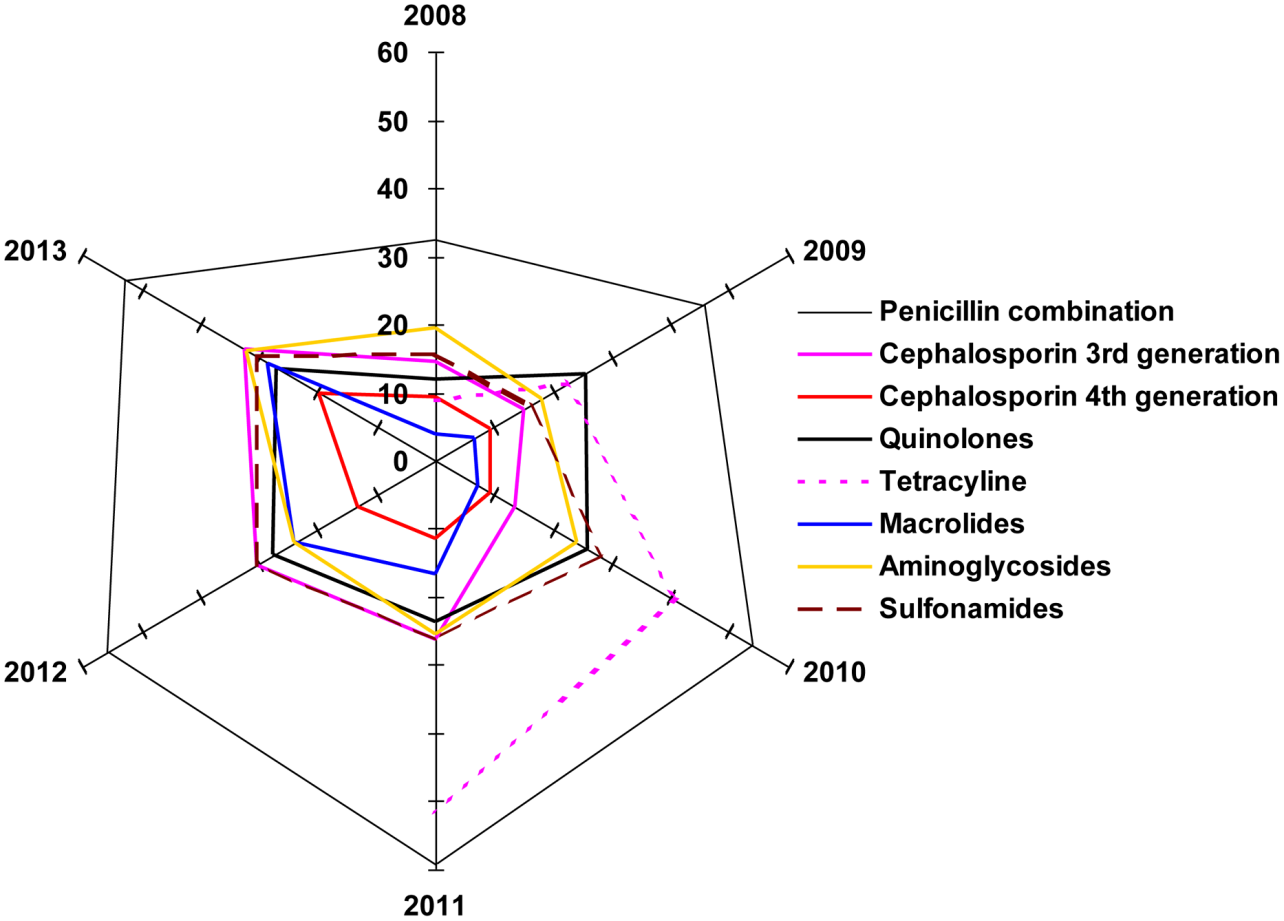
**The radar plot.** Illustration of degree of group similarity in terms of increasing resistance toward the members of the various antibiotic classes.

The radar graph shown in **Figure 6** illustrates the degree of group similarity in terms of increasing resistance toward the members of the group constituted by Penicillin combinations, fourth generation and third generation cephalosporins. Interestingly, *R*^2^ values in polynomial regression of the members of this group are 0.9958, 0.995, and 0.941 respectively. UTI pathogens exhibited high resistance toward the Penicillin combination in each year, compared to that of other drugs. Emergence of antibiotic resistance increased significantly against the Penicillin combination year after year, and it was also higher compared to that of other drugs. Macrolide-susceptible uropathogens were found in the years 2008–2010, when antibiotics resistant to Macrolides did not exceed 7.27%. Most of the multiple-antibiotic-resistant pathogens were susceptible to fourth generation cephalosporin in the years 2011–2013 (≤19.8% resistant). Uropathogens resistant to multiple classes of antibiotics also developed resistance toward quinolones, macrolides, aminoglycosides, and sulfonamides (≤32% resistant), while resistance toward the penicillin combination was found to be as high as 59.26%.

## DISCUSSION

Earlier studies from different regions of India and from other countries have reported that the most prevalent UTI pathogen was *E. coli,* followed by *Klebsiella* spp. (Francesco et al., 2007; Pires et al., 2007; den Heijer et al., 2012; Mukherjee et al., 2013; Prakash and Saxena, 2013; Renuart et al., 2013). Extended spectrum β-lactamase (ESbL)–producing organisms causing a significant increase in infection outbreaks have been reported in every continent around the world. ESbL–producing pathogens often manifest resistance determinants to several antibiotic groups like aminoglycoside and fluoroquinolones, which limit the range of effective antibiotics (Akram et al., 2007; Mukherjee et al., 2013). The incidence of ESbL-producing strains among clinical *Klebsiella* isolates has been steadily increasing over recent years and accounts for 6–17% of all nosocomial UTIs (Subha and Ananthan, 2002; Hussain et al., 2012). Drugs belonging to penicillin combination were least effective (= highest incidence of resistance) against UTI-causing *E. coli* from the year 2008 to 2013. It was shown in an earlier study that MAR enterobacterial strains, isolated from West Bengal river waters, transferred *R*-plasmids at high frequency to plasmid-less *E. coli* DH5αα strain expressing resistance to ampicillin, cephalexin (first generation cephalosporin), and cefotaxim (third generation cephalosporin; Mukherjee and Chakraborty, 2007). Maximum susceptibility was recorded for the drugs belonging to fourth generation cephalosporins. Lesser resistance to quinolones can be explained by the fact that the resistance of *E. coli* to quinolones has remained rare until recently, when the widespread use of quinolones as therapy for UTIs began (Muder et al., 1991; Aguiar et al., 1992; Llordés et al., 1993). Resistance to the penicillin combination showed a steady increase from 2008 to 2011 followed by a slight decrease in the years 2012 and 2013. As for the use of penicillin combinations (the principal components, including amoxicillin, methicillin, and piperacillin, were introduced into the Indian market from 1978 to 1989) is concerned, we have grounds to speculate that these drugs were prescribed until quinolones and third generation cephalosporin were prescribed in the treatment of UTI. It has been reported that individual exposure to a penicillin combination like co-amoxiclav raises a risk for UTIs caused by co-amoxiclav-resistant *E. coli* (Leflon-Guibout et al., 2002). It should also be noted that aminoglycosides, from gentamicin to amikacin, which were introduced within the years of 1978 to 1986, were also tried by physicians in treating UTI. Macrolides (azithromycin, clarithromycin) can have been prescribed for UTI only after they came onto the Indian market in 1992. As per current prescribing habits of physicians treating UTI, it is most obvious to speculate that doctors would sparingly prescribe a newly introduced generic extension within a subgroup or a newly introduced molecule of a group. The primary reasons are either the availability issues encountered with a newly launched product in retail outlets and/or higher prices of the newer generation drugs or later developed antibiotics. Treatment of a UTI pathogen that is resistant to all available drugs could only be possible either by using an antibiotic combination or by a single antibiotic like fourth generation cephalosporin (which appeared on the Indian scene from 2002 onwards). Cefepime, in particular, was introduced in the year 2002. The time-line of introduction of different antibiotics in India (http://www.cdsco.nic.in/forms/Default.aspx), in treating microbial infections including UTI, follows a similar pattern in development of resistance toward these drugs.

*Klebsiella* spp. from Eastern India UTI samples were maximally resistant to penicillin combination, followed by aminoglycosides and third generation cephalosporin. Studies conducted in West Bengal and around other parts of the country showed consistency in *Klebsiella* spp., which presented the second highest resistance after *E. coli*, against different classes of antibiotics; (Kothari and Sagar, 2008). Data are consistent with the findings of a northern Indian city where *K. pneumoniae* showed the highest resistance to a drug from the penicillin combination. In another study from India, which excluded the penicillin combination in determining the resistance profile of *K. pneumoniae*, it was revealed that most of the isolates were resistant to an aminoglycoside, gentamicin, followed by third generation cephalosporin, ceftriaxone and ceftazidime (Mandal et al., 2012).

Isolates obtained from this study have also shown maximum resistance to aminoglycosides and third generation cephalosporin after penicillin combination. Since penicillin combination drugs are still the choice of physicians treating *K. pneumoniae* mediated UTI infections, we observed increasing resistance against such drugs from 2008 to 2013. Increasing resistance to the penicillin combination can be explained by the fact that strains of *K. pneumoniae* are naturally resistant to ampicillin and amoxicillin, usually by the production of SHV-1, beta-lactamase encoded on the chromosome or a transferable plasmid (Nugent and Hedges, 1979).

The rate of resistance is high toward the penicillin combination and tetracycline compared to other drugs, whereas the same is less for third to fourth generation cephalosporin. The present study also indicates that the resistance to tetracycline is increasing linearly with a rate of 14.7% per year. It is also noted that the resistant capacity of bacteria to fourth generation cephalosporin is found to be below 19.8%, while that of the penicillin combination and tetracycline is found to be within the range of 8.7–59.2% and against other drugs the rates are within 3.8–32.4%.

The possible explanation of such connivance of these data on the radar plot is that the prevalence of resistance genes renders resistance to beta-lactam and or cephalosporins due to ESbL. The acquired data putatively supports this speculation, which will require molecular evidence in future studies. The resistance mechanisms to other groups of antibiotics are so varied that they are also reflected in the pattern of the individual spokes. These data also demonstrate that except tetracycline, the resistance values against all groups of antibiotics tested have seen a progressive increase at the vertices of variable years, 2011–2013.

In conclusion, the antibiotic resistance pattern of two principal UTI pathogens, *E. coli* and *Klebsiella* spp., in West Bengal, India, appears in general to be similar to that found in other parts of India, as well as other parts of the globe. Greater than 50% resistance was observed for penicillin combinations. Hence, these agents should not be used as an empiric treatment for UTI in Eastern India., Fourth generation cephalosporin and macrolides appears to be the first choice of drugor a combination of the two could be the most appropriate alternative in treating UTIs. In addition, improved record keeping and a prospective surveillance system are needed in the hospitals of Eastern India in order to facilitate regular surveillance of the occurrence of antibiotic resistance as these levels and patterns continue to change.

## Conflict of Interest Statement

The authors declare that the research was conducted in the absence of any commercial or financial relationships that could be construed as a potential conflict of interest.
